# The frequency of explosive volcanic eruptions in Southeast Asia

**DOI:** 10.1007/s00445-014-0893-8

**Published:** 2015-01-22

**Authors:** Patrick L. Whelley, Christopher G. Newhall, Kyle E. Bradley

**Affiliations:** 1 Earth Observatory of Singapore, Nanyang Technological University, Singapore, Singapore; 2 NASA Goddard Space Flight Center, Greenbelt, MD USA; 3 Mirisbiris Garden and Nature Center, Bgy. Salvacion, Sto. Domingo, Albay Philippines

**Keywords:** Global Volcano Program, Large magnitude explosive volcanic eruptions, Southeast Asia, Volcanic explosivity index, Eruption probability, Volcano morphology

## Abstract

There are ~750 active and potentially active volcanoes in Southeast Asia. Ash from eruptions of volcanic explosivity index 3 (VEI 3) and smaller pose mostly local hazards while eruptions of VEI ≥ 4 could disrupt trade, travel, and daily life in large parts of the region. We classify Southeast Asian volcanoes into five groups, using their morphology and, where known, their eruptive history and degassing style. Because the eruptive histories of most volcanoes in Southeast Asia are poorly constrained, we assume that volcanoes with similar morphologies have had similar eruption histories. Eruption histories of well-studied examples of each morphologic class serve as proxy histories for understudied volcanoes in the class. From known and proxy eruptive histories, we estimate that decadal probabilities of VEI 4–8 eruptions in Southeast Asia are nearly 1.0, ~0.6, ~0.15, ~0.012, and ~0.001, respectively.

**Electronic supplementary material:**

The online version of this article (doi:10.1007/s00445-014-0893-8) contains supplementary material, which is available to authorized users.

## Introduction

**Fig. 1:**
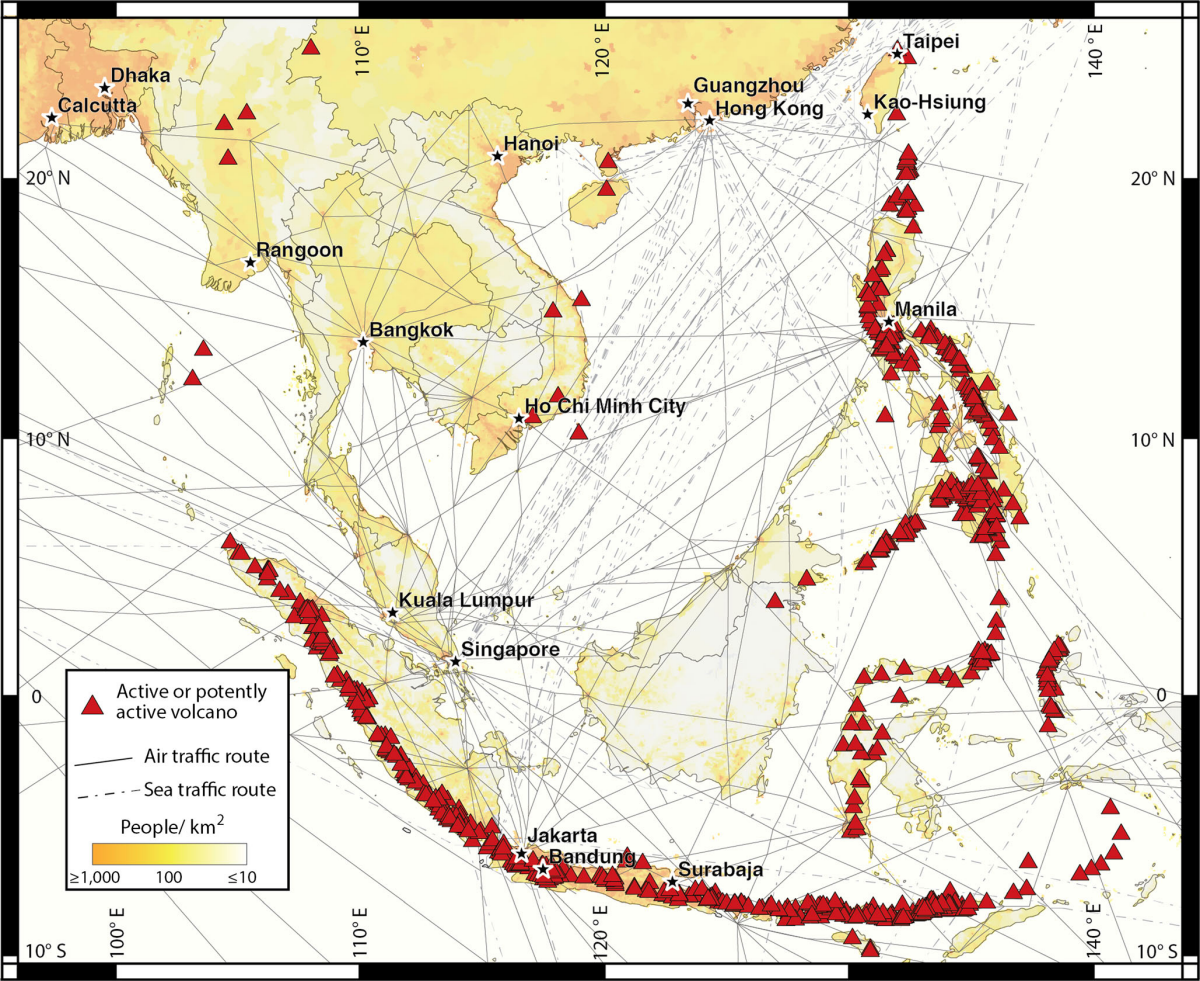
Locations of all 744 active and potentially active volcanoes in Southeast Asia (*red triangles*) and transportation pathways in the region. *Solid lines* are flight lines (after Johnson and Casadevall 1994 and Casadevall et al. 1999), *dashed lines* are cargo shipping routes (after Kaluza et al. 2010). Population data are from the Center for International Earth Science Information Network (CIESIN 2014)

Subduction at the margins of the Indo-Australian, Eurasian, and Philippine Sea Plates produces a dense arc of volcanoes in Southeast Asia, also a densely populated transportation hub (Fig. 1). Eruptions from this region include some of the most notable in human history (Table 1) making a reliable understanding of eruption frequency relevant to travel, trade, and daily life. However, the probability of eruptions from many of the volcanoes in Southeast Asia is totally unconstrained. Difficult terrain and thick vegetation discourage attempts to map many of the volcanoes on the ground. Recent advances in remote sensing enable unprecedented observation of volcano surfaces and degassing from space. We depart from previous volcano classifications (e.g., Neumann van Padang 1951; Moriya 1979, 2014; Hone et al. 2007; Siebert et al. 2010), propose a new classification scheme that considers both a volcano’s morphology and its known eruption and degassing history, and use the new classification to provide proxy data from which we make new estimates of decadal and longer term probabilities of larger volcanic explosivity index (VEI) eruptions in Southeast Asia.

**Table 1:** Notable eruptions in Southeast Asia

Volcano	Eruption year	VEI^a^	Notable
Pinatubo	1991	6	3rd largest eruption of the 20th century^b^
Krakatoa	1883	6	~35,000 killed from volcano-tsunami^c,d^
Tambora	1815	7	“The year without a summer”^e^
Rinjani^f^ (Samalas)^g^	1257	7	Caused famine in London^g^
Toba	74,000 ybp	8	Caused a human population *bottleneck* ^h^

^a^Volcanic explosivity index (Newhall and Self 1982)

^b^(Wiesner et al. 2004)

^c^(Soloviev and Go 1974)

^d^Paris et al. (2014)

^e^(Rampino and Self 1982; Wood 2014)

^f^(Nasution et al. 2003, 2004)

^g^(Lavigne et al. 2013; Newhall 2013)

^h^(Ambrose 1998)

## Methods

We compiled, from existing references, a list of all active and potentially active volcanoes within Southeast Asia. Because a large number of volcanic edifices do not appear within existing compilations, we also re-mapped the region using satellite images and digital elevation models (DEMs) to make an updated list of all active and potentially active volcanoes (Online Resource). Because eruption histories for individual volcanoes are commonly absent, we build proxy histories using records from volcanoes with similar morphology, under the assumption that volcanoes with similar geomorphic features were constructed from a similar range of eruption sizes and styles. We then combine actual and proxy histories to produce estimates of past eruption frequency and infer the spatially variable probability of future eruptions affecting the Southeast Asian region.

### Data from existing catalogues

We utilize six primary catalogues of volcano data covering our area of interest: Catalogue of the Active Volcanoes of the World Including Solfatara Fields (Neumann van Padang 1951); Data Sheets of Post Miocene Volcanoes (IAVCEI 1973); Active, Potentially Active, and Inactive Volcanoes of the Philippines, available online (PHIVOLCS 2012); Data Dasar Gunung Api Indonesia (*Translation:* Data about Indonesian volcanoes) (Badan Geologi 2011); The Smithsonian Institution’s Global Volcano Program (GVP) Database, which is a global catalogue of volcanic eruptions over the last 10,000 years (Siebert et al*.* 2010); and the large magnitude explosive volcanic eruptions (LaMEVE) database compiled by the Volcano Global Risk Identification and Analysis Project (VOGRIPA), a global database of Quaternary eruptions with volcanic explosivity index (VEI) ≥ 4 (Crosweller et al. 2012).

We combined these catalogues into one larger list of volcanoes with 825 entries. Volcanoes from the list of 825 were excluded from this study if:

1. They have summits deeper than 100 m beneath the sea. These volcanoes are, arguably, no less likely to erupt; however, the overlying water would hinder ash dispersion into the atmosphere (Kokelaar and Busby 1992), confining their potential ash hazard to the immediate surroundings. Furthermore, historical records for submarine volcanoes are virtually nonexistent and the morphologic classification scheme that we apply in this paper is inapplicable. 12 volcanoes were excluded from study due to their submersion depth.
2. Existed on multiple lists with multiple names. The location of each volcano was checked using remote sensing data, and 197 entries were found to be duplicates, either because of inconsistent spelling or the labeling of multiple peaks on the same volcano.

### Remote sensing survey

To maximize the inclusion of potentially active volcanoes in Southeast Asia in this study, it was necessary to go beyond these six catalogues. We identified previously unclassified volcanic features by close inspection of DEMs, radar scenes, and satellite imagery at a scale of 1:50,000. Geomorphic features that we take to indicate potentially active volcanic constructs include summit craters (e.g., 02.740° S, 101.855° E), arcuate escarpments representing continuous or partial caldera rims (e.g., 03.171° S, 102.177° E), isolated deep lakes or large semi-circular depressions not associated with faults (e.g., 00.031° N, 100.496° E), flow-like morphology (e.g., 02.740° S, 101.855° E), and conical edifices with radial drainage patterns (e.g., 04.986° S, 104.588° E). It is likely that this approach is insufficient to identify all hazardous volcanic features in the study area. Furthermore, we do not estimate the frequency of formation of new volcanoes. On the other hand, because stratovolcanoes can lie dormant for very long periods before again becoming active (e.g., Connor et al. 2006), we include deeply incised, radially symmetric edifices that do not preserve other morphology indicating active volcanism, some of which may be truly extinct. Our mapping criteria would have identified the ~4 km diameter caldera of Chaitén, Chile as a potential source of regional ashfall hazard prior to its 2008 eruption, while it sat unmonitored (Carn et al. 2009). An example of a similar feature identified by our mapping but not listed in a regional database is Liu volcano, located just southeast of Ranau caldera (05.029° S, 104.002° E). This survey resulted in the identification of 128 additional volcanoes. We assign a name to each of these features using the GeoNames Database (2013).

The resulting catalogue contains 744 volcanoes we consider to be active or potentially active (Fig. 1 and Online Resource). Our criterion for potentially active is a volcano that is morphologically youthful (i.e., exhibits easily discernable primary volcanic features such as flows or craters), but its capacity for future eruptions is unknown. Active volcanoes are listed as such on an existing catalogue. Both active and potentially active volcanoes are located in the Philippines, Indonesia, Myanmar, Taiwan, the southeast coast of Vietnam, and the Hainan province of China.

### Proxy eruption histories

Of the 744 active or potentially active volcanoes in Southeast Asia, approximately 70 have erupted within the past 100 years. Some of these 70 have been mapped in some detail. However, the eruptive histories of 90 % of the volcanoes in Online Resource are poorly constrained or totally unconstrained. While field studies of each volcano are necessary to accurately assess their eruptive histories, morphologic observations made using remote sensing data can also provide general information about past activity (e.g., Hulme 1974; Moriya 1979; Greeley 1982; Whelley et al. 2014; Grosse et al. 2014). The shape of a volcano and its collection of deposits are the result of all previous eruptions and periods of erosion throughout the volcano’s history. By assuming that volcanoes with similar morphology have had similar recent eruptive histories, we can group volcanoes by morphology and use the eruption histories of well-studied examples within each morphologic category as proxy histories for the other, similar looking, volcanoes in the class. Volcanoes with ≥3 eruptions (or large caldera type volcanoes with ≥1) in the LaMEVE and/or GVP databases are used to build proxy histories. We refer to them as well-studied for simplicity, but note that even these 70 volcanoes require more detailed mapping to be adequately understood. This approach is not well suited for forecasting the behavior of a particular volcano; rather, it is used to understand the regional probability of volcanic eruptions.

### Volcano classification

**Fig. 2:**
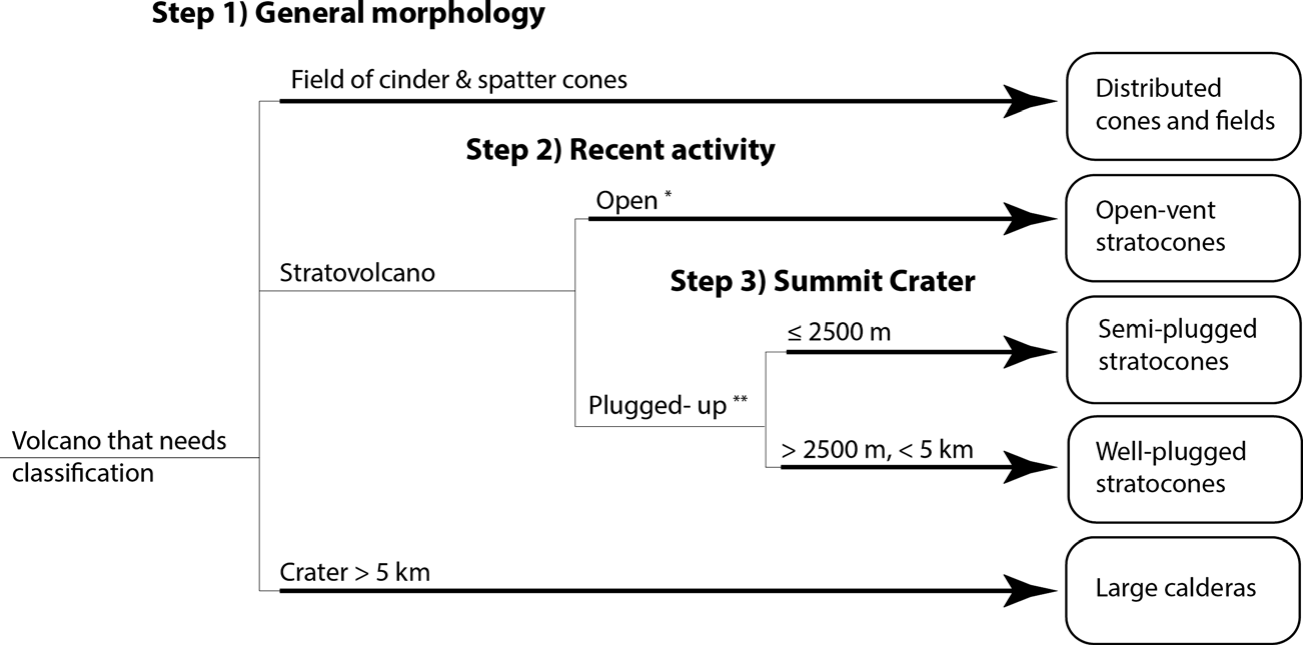
Volcano classification flowchart that illustrates the three-step classification scheme we used to differentiate volcanoes by morphology (step 1 and 3) and recent eruption activity (step 2). In step 2, stratovolcanoes that have >2 VEI 1 or 2 eruptions in past decade and average >8 emission events per year of 0.8 kt/day of SO_2_ since 1979 as tabulated by Aerocom and collected by TOMS, COSPEC, and ONEMI and classified as open-vent stratocones. Plugged-up stratocones have not erupted in the past decade or have insufficient SO_2_ emissions

Using remote sensing data and eruption records (where available), we associated each volcano in our database with one of five classes in three steps (Fig. 2). In the first step, volcano-geomorphic features were identified by visual inspection. Image data used include Landsat, GeoEye, and DigitalGlobe images acquired through Google Earth Professional and Advanced Spaceborne Thermal Emission and Reflection Radiometer (ASTER) images acquired through NASA and Jet Propulsion Laboratory’s ASTER Volcano Archive. TerraSAR-X Radar scenes were acquired from Deutsches Zentrum für Luft- und Raumfahrt e.V. (DLR) through a data archive access grant. For topographic data, we used a derivative of Shuttle Radar Topography Mission (SRTM) data, acquired through Google Maps, Open Street Map, and QGIS.

**Fig. 3:**
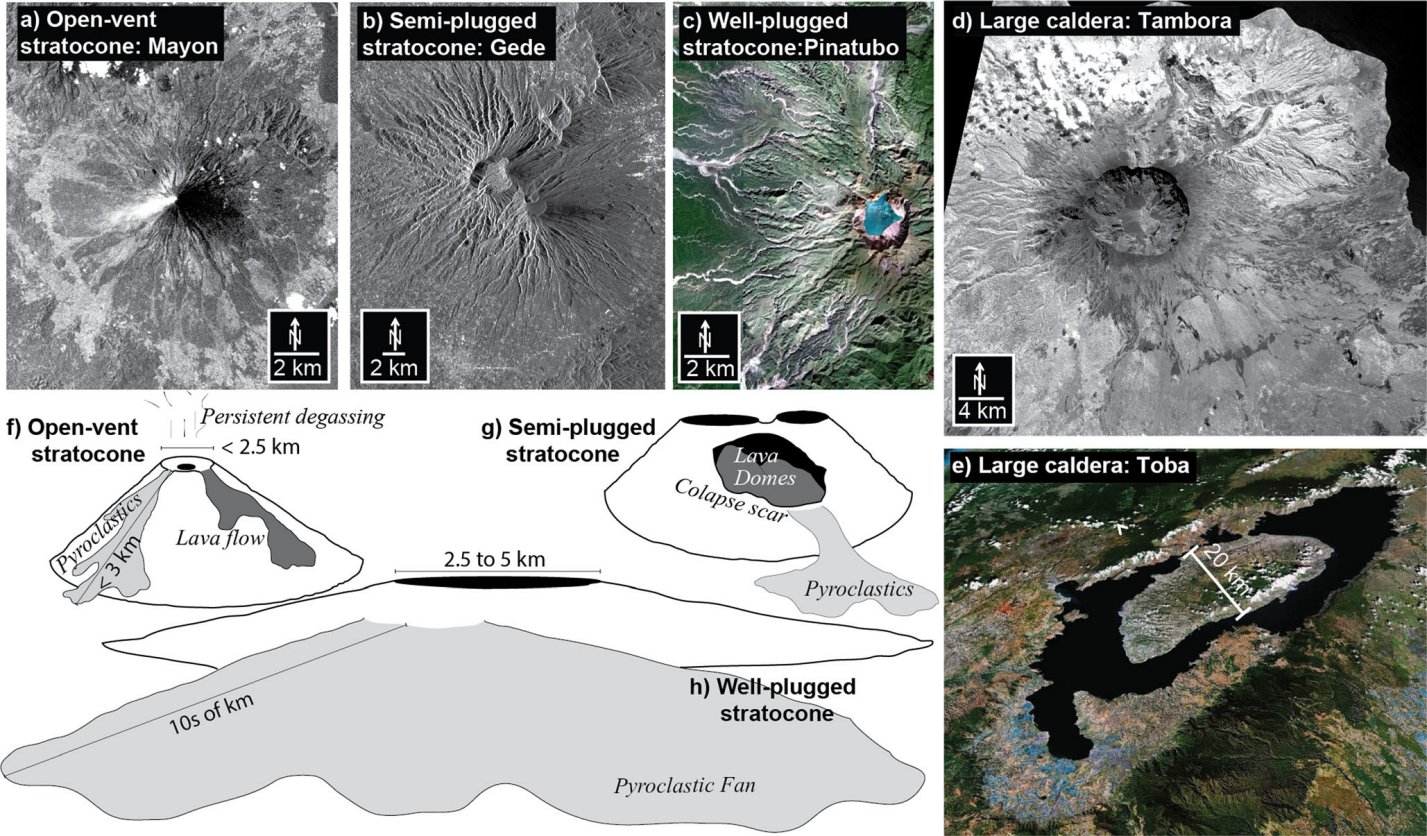
Volcano class examples and details. **a** Mayon volcano (an open-vent stratocone), photograph from NASA/STS083, **b** Gede volcano (a semi-plugged stratocone) in Terra SAR-X scene: TSX1_SAR__MGD_RE___SC_S_SRA_20100218T110851_20100218T110913, **c** Pinatubo volcano (a well-plugged stratocone), image from DigitalGlobe and GeoEye, **d** Tambora volcano (a large caldera), NASA/ASTER image L1B_20030729022755_3B (in the visual spectrum), **e** Toba Caldera (a large caldera) oblique visualization of DigitalGlobe images and NASA/SRTM topography from Google Earth. Notice the variety of remote sensing data types that are useful for identifying volcanic features. Idealized sketches of **f** an open-vent stratocone, **g** semi-plugged stratocone, and **h** well-plugged stratocone, show key morphologic features of the volcano classes

For each volcano, we measured the long axis of the summit crater and the lateral extent of the volcanic apron (Fig. 3) and noted the presence of lava flows, pyroclastic, or debris avalanche deposits. We also identified cones, calderas, collapse scars, and domes. Based on this morphologic assessment, volcanoes are placed into one of three categories. Collections of small (≤1 km diameter) volcanic centers and lava flows are classified as distributed cones and fields. Volcanoes with a crater ≥5 km are classified as large calderas (following: Newhall and Dzurisin 1988). Volcanoes that remain are stratovolcanoes and are further differentiated in the following steps.

In the second step, because volcano monitoring has improved significantly, we perform an assessment of the past several decades of eruptive activity. We considered a stratovolcano to be an open-vent stratocone if it produced persistent gas plumes (observable in satellite remote sensing and catalogued by AeroCom [Diehl 2009] following Andres and Kasgnoc 1998) and small (VEI 1 or 2) eruptions approximately annually or more often. In contrast, a stratovolcano that erupts less frequently and traps most of the gas supplied to its reservoir in between eruptions (Newhall 2007; Rose et al. 2013) is termed a plugged stratovolcano. Because a few volcanoes change their eruptive and degassing behavior from time to time, our classification of a volcano is for the foreseeable future but not necessarily forever.

The third step returns to morphology as a proxy for several additional criteria. Plugged stratovolcanoes are further differentiated into semi-plugged and well-plugged based on frequency and VEI’s of eruptions and width of pyroclastic apron, all represented in classification by a single parameter: summit crater long axis diameter. Implicit in designation of the well-plugged category is a common association of summit crater size 2.5–5 km with eruptions of VEI 6, repose periods in hundreds of years, wide pyroclastic aprons and, from Pinatubo, accumulation of a large mass of gas, far in excess of saturation, probably accumulated over centuries. The semi-plugged category is intermediate between well-plugged and open-vent stratocones—in VEI’s and repose periods—and also has many examples of volcanoes with summit dome complexes and sector collapses. The size of a summit crater or caldera correlates with the size of the largest recent eruptions of a volcano (Smith 1979), hence, our use of crater diameter as a proxy for explosivity. Most semi-plugged stratocones (like Kelut or Gede) have summit craters ≤2500 m while most well-plugged stratocones (like Pinatubo) have summit craters between 2500 and 5000 m.

For clarity, Fig. 2 is a flowchart of the classification process, and the paragraphs that follow include more details of each of the five classes of volcanoes that are common in Southeast Asia.

**Fig. 4:**
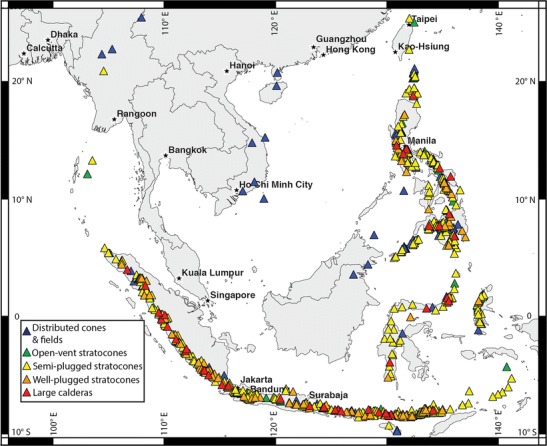
A map of active and potentially active volcanoes of Southeast Asia. *Colors* indicate the volcanoes’ classification

Distributed cones and fields are maars, cinder cones, spatter cones, and shields or fields of multiple constructs. An example is the Laguna Volcanic Field, Philippines (13.204° N, 123.525° E). There are 130 distributed cones and fields in the study area. We count stratovolcanoes within volcanic fields separately from the field (e.g., Lamongan Maar Field is counted as a distributed field while Lamongan stratovolcano is counted as a semi-plugged stratocone: 07.979° S, 113.342° E) (Fig. 4).

Open-vent stratocones (Newhall 2007; Rose et al. 2013) are persistently degassing, frequently active (a few years or less between eruptions), steep-sided stratocones. They typically have small summit craters, spatter ramparts, small pyroclastic fans (typically <3 km but up to 5 km head to toe), and lava flows that originate from the flanks or the summit. Mayon Volcano, Philippines (13.257° N, 123.685° E) is an open-vent stratocone, and there are 60 such volcanoes in the study area (Fig. 4).

Semi-plugged stratocones are composite cones with decades between eruptions and commonly with dome complexes, pyroclastic fans, and/or debris avalanche deposits or collapse scars. Their summit craters are ≤2500 m. Kelut Volcano, Indonesia (07.933° S, 112.308° E) and Gede Volcano, Indonesia (06.7833° S, 106.9833° E) are two of the 411 semi-plugged stratocones in the study area; most volcanoes in the region belong to this class (Fig. 4).

Well-plugged stratocones typically have centuries or longer between eruptions, extensive (tens of km) pyroclastic fans and summit craters or calderas 2500 to 5000 m in diameter. Some fumarolic activity may occur, but most gas supplied from depth during repose periods accumulates in the magma and then powers a large, VEI 6 explosive eruption. Pinatubo Volcano, Philippines (15.133° N, 120.350° E) and Parker Volcano, Philippines (06.113° N, 124.891° E) are well-plugged stratocones, and there are a total of 102 in the study area (Fig. 4).

Large calderas are volcanic depressions of collapse origin with long axes >5 km. Typically, they are surrounded by expansive ignimbrite sheets, and many have central domes. Tambora Volcano, Indonesia (08.25° S, 118.00° E) and Toba Caldera, Indonesia (02.583° N, 098.833° E) are classic examples. We include in this category a few silicic dome complexes that might eventually produce large caldera-forming eruptions as part of this class, e.g., Rikit Dekat (03.988° N, 097.278° E). There are 41 large calderas in the study region (Fig. 4), of which some have proximal stratocones or fields of maars (e.g., Taal and Toba). These adjacent centers are counted separately from the associated caldera.

Note that the classification given here represents the volcanoes’ current morphology and latest activity. Some volcanoes migrate from class to class during their lifetime.

## Eruption frequency

**Fig. 5:**
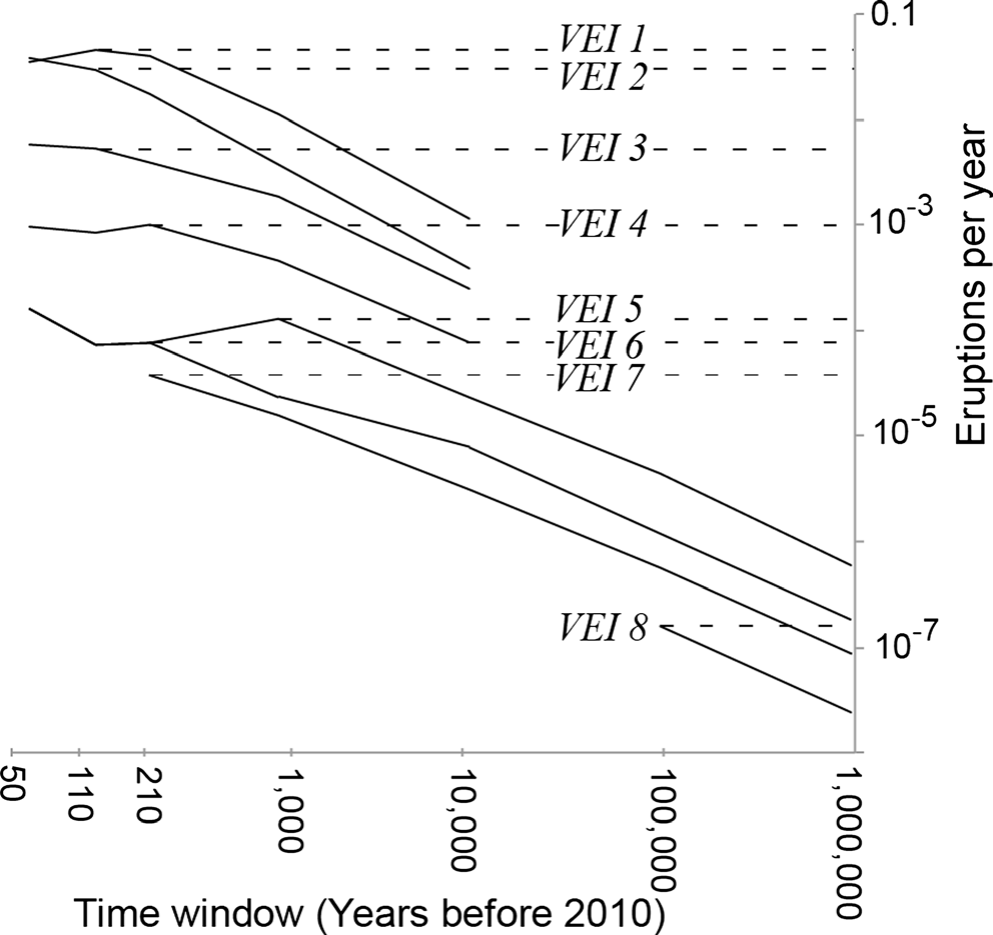
Apparent eruption frequency (*f*_*e*_), solid lines calculated at 7 time intervals using eruption statistics from Japan and Southeast Asia. The decrease in eruption frequency over time illustrates incompleteness of the data. *Dashed lines* represent an approximation of the actual eruption frequency (*F*_*e*_)

We use Holocene eruption statistics from the Smithsonian Institution GVP (Siebert et al. 2010), and Pleistocene eruption statistics from LaMEVE (Crosweller et al. 2012). Apparent eruption frequency (*f*_*e*_) is determined by:

1 $$ {f}_e=\frac{n_e}{t} $$

where *n*_*e*_ is the number of known eruptions and *t* is the interval of time (in years) between the oldest eruption and the year 2010 (the year of the youngest eruptions in the catalogue when the study began). Deposits from small eruptions (VEI ≤ 3) are not well preserved and are consequently under-represented in portions of eruption databases that rely on the geologic record (i.e., pre-historic). Large eruptions (VEI ≥ 5) are more completely represented in the geologic record (Siebert et al. 2010) but can have 1000 year or longer recurrence intervals and are therefore under-represented in portions of eruption databases that rely on the historical record. To ensure that eruption frequencies are not underestimated, we used different values of *t* to evaluate the frequency of each eruption size (Table 2; Fig. 5). We estimate the actual eruption frequency (*F*_*e*_) for each VEI with Eq. 1 using the value for *t* that balances the repose between eruptions and the completeness of the eruption record. This *t* is approximated by finding the maximum of the *f*_*e*_ curve (Fig. 5).

**Table 2:** Time intervals used to find *F*_*e*_

Eruption explosivity	Time interval *t* (years)	Calendar years
VEI 1	50	1960–2010
VEI 2	110	1900–2010
VEI 3	110	1900–2010
VEI 4	210	1800–2010
VEI 5	1000	
VEI 6	10,000	
VEI 7	10,000	
VEI 8	100,000

**Fig. 6:**
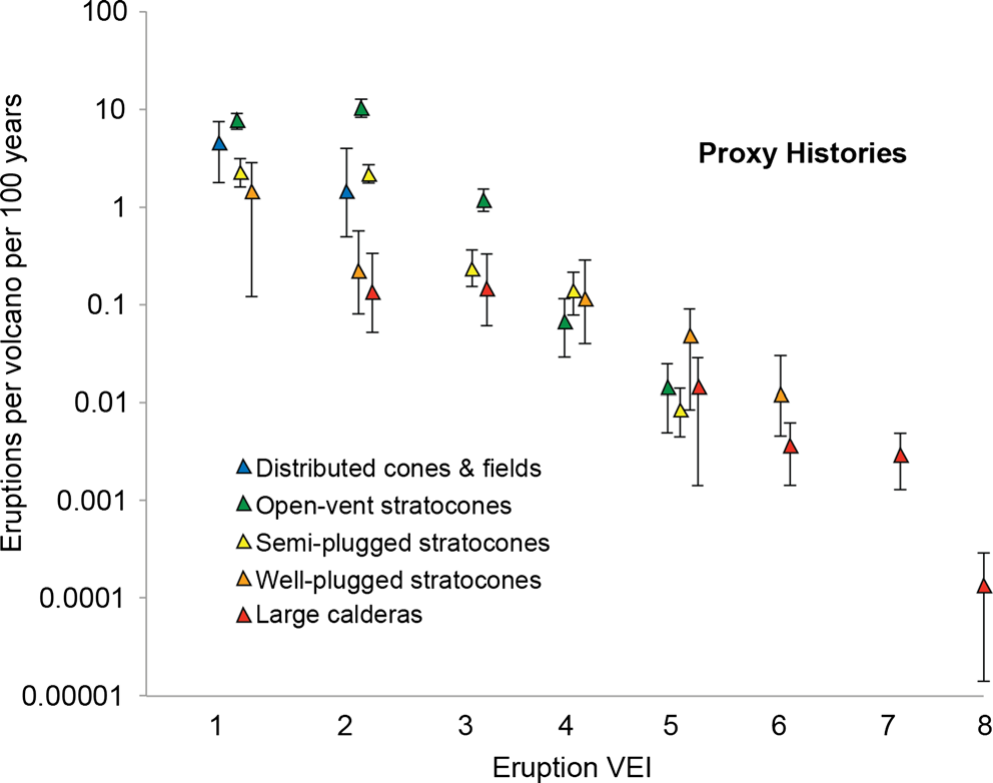
VEI profile (frequency of eruptions per volcano per 100 years vs. VEI) for a typical volcano within each volcano morphologic class. *Whiskers* represent 90 % confidence interval standard error about the mean frequency. These same data are shown in Table 3

We averaged eruption frequencies (*F*_*e*_) at well-studied volcanoes within every volcano class, for each VEI, to determine each class’s *typical behavior*. To increase our sample size and to utilize the excellent historical and geological record of volcanism in Japan, we used eruption statistics from Japanese volcanoes with ≥3 catalogued eruptions (or ≥1 for large calderas) to supplement eruption histories in all classes. Our resulting eruption profiles (Table 3; Fig. 6) are based on 70 Southeast Asian and 55 Japanese volcano histories.

**Table 3:** Eruption profiles for each volcano class and a comparison of regional eruption frequency calculated here with two eruption databases

Eruption explosivity	Eruptions per 100 years per volcano for each volcano class (*f*_*e*_ × 100)					Eruptions per 100 years in all SE Asia		
	Distributed cones and fields (*n* = 130)	Open-vent stratocones (*n* = 60)	Semi-plugged stratocones (*n* = 411)	Well-plugged stratocones (*n* = 102)	Large calderas (*n* = 41)	This work	GVP^a^	LaMEVE^c^
VEI 1	4.7	7.7	2.3	1.5	0	2163	302	No data
VEI 2	1.5	10	2.3	0.23	0.14	1814	443	No data
VEI 3	0	1.2	0.24	0	0.15	182	49	No data
VEI 4	0	0.071	0.14	0.12	0	76	10	10
VEI 5	0	0.015	0.009	0.050	0.015	9.97	1.4	1.6
VEI 6	0	0	0	0.013	0.0038	1.45	0.06	0.1
VEI 7	0	0	0	0	0.0031	0.13	0.5^b^	0.5^b^
VEI 8	0	0	0	0	0.00015	0.01	No data	0.001

^a^Calculated using Eq. (1) and all eruptions in SE Asia included in the GVP database (Siebert et al. 2010) within the time intervals given in Table 2

^b^The 1815 eruption of Tambora is the only VEI 7 eruption in the time interval *t* in either database in Southeast Asia

^c^Calculated using Eq. (1) and all eruptions in SE Asia included in the LaMEVE database (Crosweller et al. 2012) within the time intervals given in Table 2

While eruption histories from all of our classes follow a similar pattern of exponential decay, with small eruptions being more common than larger ones, there is some notable variability in eruption characteristics between classes. In the study area, distributed cones and fields only produce VEI 1 and 2 eruptions. Open-vent stratocones have frequent VEI 1, 2, and 3 sized eruptions and infrequent VEI 4 eruptions. Semi-plugged stratocones produce less frequent eruptions, up to about VEI 5, presumably due to their less open plumbing and longer repose. Well-plugged stratocones produce eruptions up to VEI 6. Large calderas are capable of VEI 7 and even VEI 8 eruptions; the latter of which has not occurred on Earth since Toba’s 74 ka eruption (e.g., Rose and Chesner 1987; Chesner 2012).

The impact of our approach, using eruption histories from well-studied volcanoes to build proxy histories for other volcanoes, is evident in Table 3. By accounting for volcanoes that are potentially active as well as those that are known to be active, we aim to overcome incomplete geological and historical eruption records in Southeast Asia. As this approach might overestimate eruption frequency, we test for bias in section 3.1.

### Over estimation tests

Eruption statistics for volcanoes in Southeast Asia exist for ~70 volcanoes that have erupted since 1800. It was on this year that in present day, Indonesia, the trade organization, the Dutch East India Company was nationalized forming the Dutch East Indies, a colony of The Netherlands (Dick et al. 2002). Historical records of earthquakes and volcanic eruptions are more numerous and arguably more reliable after 1800. Even so, 90 % of the volcanoes in Online Resource have been historically quiet since 1800. Proxy histories are derived in large part from volcanoes that have erupted since 1800. However, assigning proxy histories derived from historically erupting volcanoes to historically quiet ones potentially overestimates eruption frequency of the latter. To check for this bias, we use two different tests. In the first test, we use two samples of volcanoes from Japan, where the historical and geological records are robust for ~2000 years, and for the second, we use statistical measures to analyze periods of inactivity preceding VEI ≥ 4 eruptions.

**Fig. 7:**
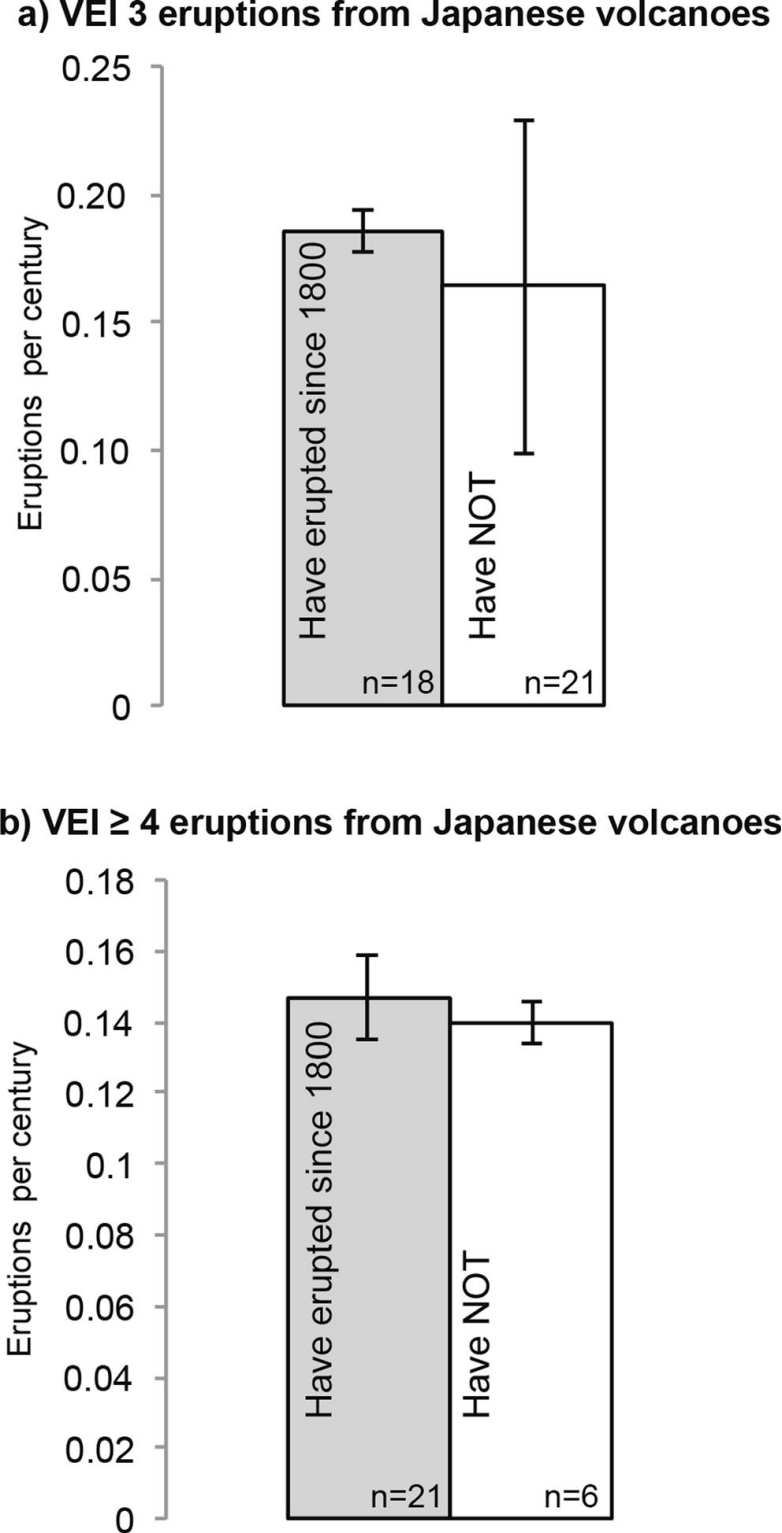
Comparison of average Japanese volcano eruption frequencies (in eruptions per century) for eruptions from year 0 to 2010. *Gray bars* represent volcanoes that have erupted since 1800 and *white bars* are volcanoes that have not. “*n*” values indicate the number of eruptions represented in each bar. The *upper diagram* (**a)** is for VEI 3 eruptions; the *lower diagram* (**b)** is for VEI ≥ 4 eruptions. *Standard deviation whiskers* indicate the uncertainty of estimates and thus statistical significance of any difference in histogram box height. In both comparisons (**a** and **b**), the whiskers overlap indicating that the samples are statistically indistinguishable

In the first test, one sample is made up of Japanese volcanoes that have erupted since 1800; the other includes Japanese volcanoes that have not erupted more recently than 1800. If there is a significant difference between the eruption frequency of the two (Fig. 7) for the past 2000 years, then there is a bias and we must adjust our Southeast Asian eruption probabilities. We test this using the eruption frequency mean and standard deviation of each sample.

The eruption frequencies of the two samples of Japanese volcanoes are within a standard deviation of each other indicating that they are statistically indistinguishable. This suggests that using historical eruption frequencies of VEI ≥ 3, even for volcanoes that have been long quiet, does not overestimate our overall probabilities of eruptions. Therefore, in Southeast Asia, proxy histories built with VEI ≥ 3 eruptions from historically erupting volcanoes should approximate the activity of historically quiet volcanoes. Insufficient data exist for VEI ≤ 2 eruptions before 1800 to similarly test for a bias in smaller eruptions. Because VEI ≤ 2 eruptions commonly have only local impact, we ignore them for the further purposes of this study.

**Fig. 8:**
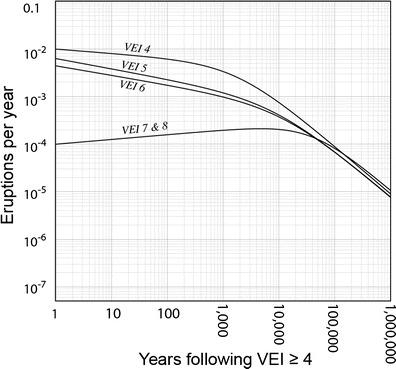
Age-specific eruption rates for VEI 4–8 eruptions derived from 142 eruption records. Rates for VEI 7 and 8 are added together. The hazard function for VEI 4 decreases sharply after 1000 years of repose; that for higher VEI’s decreases sharply after 10,000 years

In the second test for bias, we use “survival analysis” which is commonly used in medical and engineering studies. In these other fields, the time between medical treatment and the return of symptoms or between manufacture of a part and its failure are of interest. We employed survival analysis to see how long a volcano must be quiet before being considered “dead” (following Connor et al. 2006). To do so, we used eruption statistics from Southeast Asia and Japan for eruptions with VEI ≥ 4. Eruption statistics from this VEI range are available in both LaMEVE, and the GVP and the former include eruptions into the Quaternary. We placed the number of years between a VEI ≥ 4 eruption and a subsequent VEI 4, 5, 6, and ≥7 eruptions in rank order and found a best-fit log-logistic survival function curve. This was done for VEI 4, 5, 6, and ≥7 eruptions separately. The log-logistic distribution fits the eruption frequency data well and accommodates extreme values that would be outliers in an exponential distribution. Survival functions are related to probability distribution functions and indicate the probability that the variate (in this case, a volcanic eruption) will follow a period of time (repose interval) that exceeds a given time (*T* time in years). Hazard functions are the ratio of the probability density function and survival function and indicate an age-specific failure rate (or regional eruption rate, in this case). Figure 8 shows the hazard functions for VEI 4, 5, 6, and ≥7 eruptions.

Following a VEI ≥ 4 event, the rate of subsequent VEI 4 eruptions drops only slightly during the first 1000 years after an eruption and then falls off sharply. The rate of subsequent VEI 5 and 6 events follows a similar pattern but decreases sharply after 10,000 years. In contrast, eruption rates gradually increase for VEI 7 and 8 eruptions to a maximum ~10,000 years after a VEI ≥ 4 event, probably because they need millennia to re-accumulate enough volcanic gas to drive a giant VEI 7 or 8 eruption. Thus, stratovolcanoes (open-vent, semi-plugged, and well-plugged stratocones) that have not been asleep for at least 10,000 years remain potentially active while large calderas sleep longer before VEI 7 or 8 eruptions. A dedicated mapping and dating effort is needed to determine the age of the youngest deposits from the majority of the volcanoes in Southeast Asia. Even so, it is clear from the survivor function analysis that the time between VEI ≥ 4 events is commonly orders of magnitude longer than the historical record in the region, further suggesting that our approach does not introduce significant bias to regional eruption frequency.

### Eruption probability

For a regional probability estimate, we assume a Poisson distribution to evaluate the probability of an event (e.g., an eruption) occurring in a given time interval (Sheskin 1997). The probability (Pr_*e*_) of an eruption occurring within the next *y* years is estimated by:

2 $$ \Pr {}_e=1-{e}^{-y/{F}_e} $$

**Fig. 9:**
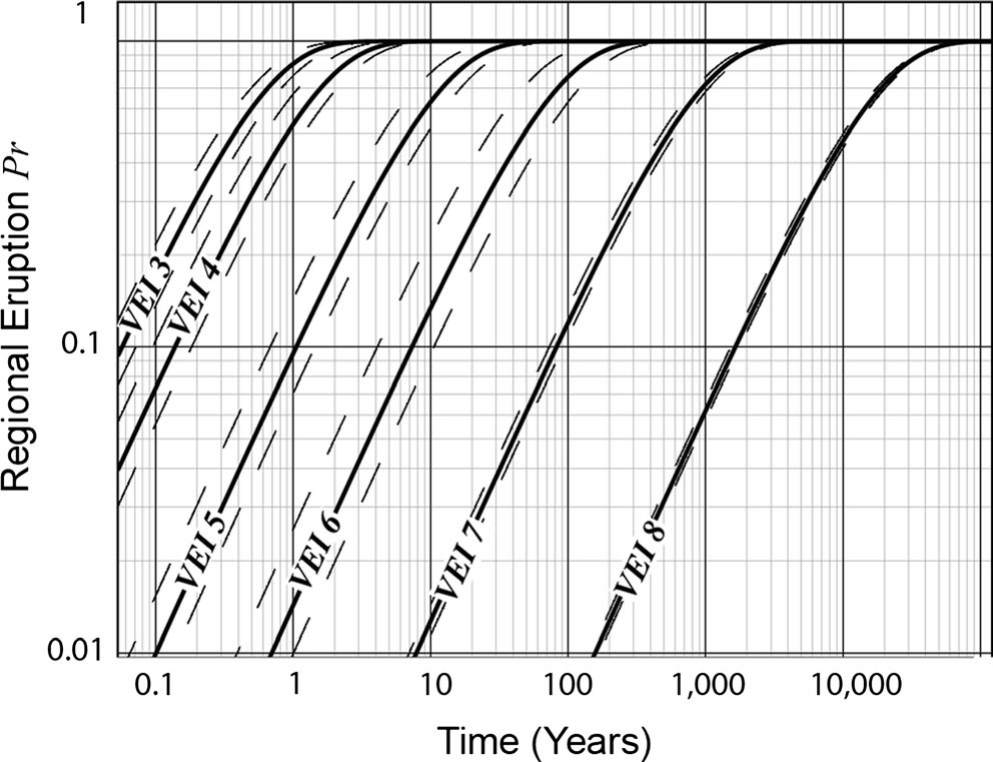
Time-dependent eruption probability curves for Southeast Asia (*solid lines*) and 95 % confidence interval standard error (*dashed lines*)

Using Eq. (2), we calculated the probability of VEI 3 through 8 eruptions in Southeast Asia (Fig. 9 and Table 1). Given here are a few examples from the graph and the standard error (in parentheses). VEI 3 and 4 eruptions are virtually certain to occur someplace in the region in the next decade, while there is a 0.6 (0.5 to 0.8) probability of a VEI 5 eruption in the same period. Larger eruptions are far less likely to occur. A VEI 6 eruption (similar in size to the 1991 Pinatubo eruption) has a 0.12 (0.09 to 0.2) probability in a decade while a VEI 7 eruption (similar in size to the Tambora 1815 eruption) has a 0.12 (0.11 to 0.13) probability in a century. A VEI 8 eruption (similar in size to a Toba 74 ka event) has a 0.06 (0.05 to 0.07) probability in a millennium.

## Discussion

We present a classification scheme made using Japanese and Southeast Asian volcanoes. However, the volcanic characteristics that define each category are not unique to these regions. For example, Llaima and Villarrica Volcanoes, Chile and Mt Etna, Italy are examples of open-vent stratocones outside Southeast Asia. Soufriere Hills, Montserrat, and Mount St. Helens, WA-USA are examples of semi-plugged stratocones. An updated global classification of stratocones is needed and would provide a baseline for comparison of volcanic behavior. Recent work demonstrates that there is an abundance of quantitative information in volcanic morphology that can be extracted from remote sensing data (e.g., Thouret and Németh 2012 and references therein). Grosse et al. (2014) systematically measured many morphometric parameters of hundreds of stratocones, but stop short of proposing a global classification scheme, as the authors found insufficient differences between groups of volcanoes. We have shown that combining morphology and volcanic unrest characteristics illuminates important differences between types of volcanoes and their eruptive activity. Future work will build on the classification scheme described in this paper and incorporating more quantitative geomorphology, to classify the rest of the volcanoes of the world.

Volcano monitoring equipment is presently concentrated on a small number of active volcanoes that are known to pose significant eruption hazards. Comparisons of existing instrumentation vs. estimated eruption probability for individual volcanoes in our database could inform volcano observatories and development aid organizations about where monitoring gaps exist and may need to be filled in the future.

## Summary

1. There are ~750 active and potentially active volcanoes in Southeast Asia. Detailed mapping efforts are needed to assess the volcanic histories of most of them.
2. Volcano morphology is used here to group volcanoes and build proxy eruption histories for inadequately studied volcanoes and to determine the probability that a quiet volcano is extinct.
3. The incompleteness of the geologic record is overcome, when investigating eruption frequency, by calculating the apparent eruption frequency only for time intervals of relatively complete reporting.
4. Open-vent stratocones and semi-plugged stratocones erupt more often but less explosively than well-plugged stratocones and large calderas.
5. The probabilities of VEI 3–8 eruptions somewhere in Southeast Asia in a decade are VEI 3 and 4, nearly 1.0; VEI5, ~0.6; VEI 6, ~0.15; VEI 7, ~0.012; VEI 8, ~0.001.

## Electronic supplementary material

Below is the link to the electronic supplementary material.

Online Resource. Active and potentially active volcanoes of Southeast Asia. This table is sorted alphabetically by volcano name, column C. Column A is a number assigned to each volcano for this study, while column B is the more widely used Smithsonian Global Volcano Program Volcano Number. Note that many of the volcanoes in this list are not on the GVP list and so do not have GVP numbers. Column D lists other names given to the same volcano or sub features. Volcano locations (Column E and F) are given in decimal degrees using the WGS 84 datum and elevations (column G) are in meters. Volcano classes are identified for each volcano in column H. Column I indicates whether the eruption histories were implied from volcano specific studies or from the ‘well studied’ volcanoes of the class. Columns J through Q list the number of eruptions per year for each volcano. In Columns R through U, ‘X’ marks the catalogues that include each volcano. (XLSX 124 kb)
